# Type II diabetes and cognitive function among older adults in India and China—results from Harmonized Cognitive Assessment Protocol studies

**DOI:** 10.3389/fpubh.2024.1474593

**Published:** 2024-11-06

**Authors:** Subidsa Srikantha, Jennifer Manne-Goehler, Lindsay C. Kobayashi, David Flood, Silvia Koton, Alden L. Gross

**Affiliations:** ^1^Department of Epidemiology, Johns Hopkins Bloomberg School of Public Health, Baltimore, MD, United States; ^2^Division of Infectious Diseases, Massachusetts General Hospital, Harvard Medical School, Baltimore, MA, United States; ^3^Institute for Healthcare Policy and Innovation, University of Michigan, Ann Arbor, MI, United States; ^4^Department of Epidemiology, University of Michigan School of Public Health, Ann Arbor, MI, United States; ^5^Department of Internal Medicine, University of Michigan, Ann Arbor, MI, United States; ^6^Stanley Steyer School of Health Professions, Faculty of Medicine, Tel Aviv University, Tel Aviv, Israel; ^7^Department of Mental Health, Johns Hopkins Bloomberg School of Public Health, Baltimore, MA, United States

**Keywords:** cognitive function, diabetes, harmonization, rural–urban, education, epidemiologic transition, nutrition transition

## Abstract

**Objective:**

Type II diabetes is a recognized risk factor of declining cognitive function in high-income countries. However, there is limited research on this association across low- and middle-income countries. We aimed to examine and compare the relationship between type II diabetes and cognition amongst adults aged 60 years and older for two of the largest LMICs: India and China.

**Methods:**

Cross-sectional data was analyzed from population-based Harmonized Cognitive Assessment Protocols studies in India (*n* = 4,062) and China (*n* = 9,741). Multivariable-adjusted linear regression models examined the relationship between diabetes (self-reported or biomarker HbA1c ≥6.5%) and general cognition. Interaction testing assessed effect modification based on urban versus rural residence and educational attainment.

**Results:**

Type II diabetes was not associated with general cognitive scores in India or China in fully adjusted models. Interaction testing revealed a positive association in rural but not urban residences in India, however this was not seen in China. Both countries showed effect modification by education attainment. In India, diabetes was associated with higher average cognitive scores among those with none or early childhood education, while the relationship was null among those with at least an upper secondary education. In China, diabetes was inversely related to average cognitive scores among those with less than lower secondary education, while the relationship was null among the remainder of the study sample.

**Conclusion:**

The type II diabetes and cognitive function association in India and China differs from that observed in high-income countries. These findings suggest epidemiologic and nutrition transition variations. In India, health care access, urbanization and social differences between urban and rural areas may influence this relationship. In both countries, epidemiologic and nutrition patterns may adversely impact individuals from socially and financially vulnerable populations with less than lower secondary education. Longitudinal research using harmonized cognitive scores is encouraged to further investigate these findings.

## 1 Introduction

With the global population aging at a substantial and increasing pace, dementia has become a leading cause of morbidity and mortality (1). As of 2019, approximately 55.2 million people worldwide were living with dementia, of whom 60% were living in low- and middle-income countries (LMICs) (1, 2). This estimate is projected to rise to 139 million people by 2050 (1). Reducing or treating modifiable risk factors by 10% per decade until 2050 may prevent approximately 8.8 million cases of dementia, underscoring the importance of addressing potentially preventable dementia risk factors that can lead to paths for primary prevention initiatives (3, 4).

Type II diabetes is a recognized risk factor for cognitive decline and dementia in older adults (5–7). Findings from prospective observational data in high-income countries have reported a 56% elevated risk of dementia among those diagnosed with type II diabetes (7). There are several mechanisms which may underlie the association between type II diabetes and dementia, including neurotoxicity resulting from hyperglycemia, alterations in amyloid metabolism, increased risk of cerebrovascular disease, and a combination of these mechanisms (6). Additionally, insulin resistance, which is characteristic of type II diabetes, has been shown to be a risk factor of dementia via a surge of generating amyloid-beta in the brain (8). The relationship between diabetes and declining cognitive health is multifaceted and complex, thus further research is needed to understand how this risk factor may differ outside of high-income countries.

There is little research on the relationship between diabetes and dementia across LMICs such as India and China; two of the most populous countries in the world (9, 10). Diabetes is highly prevalent among these countries. India is estimated to have a population of 74.2 million people with diabetes (11) while China has an estimated population of 141 million with diabetes (12). Furthermore, as socioeconomic growth and industrialization occur at a rapid pace in these developing LMICs, epidemiologic and nutrition transitions may drastically influence shifts in health behaviors and outcomes that could deviate from what is observed in Western, high-income countries (13).

Epidemiologic transition encompasses population shifts in causes of mortality and morbidity from communicable diseases (driven by malnutrition and poor maternal conditions) to noncommunicable/chronic diseases, including type II diabetes (14). It has multifactorial implications for disease and health trends (14, 15). Drivers of this trend include increased life expectancy, urbanization, globalized food production, economic growth, and unhealthy lifestyles (such as physical inactivity and poor diet) (16). Nutrition transition also plays a role in driving excessive intake of nutrient poor foods through changes in food availability and increased purchasing power (14). Consequently, nutrition patterns shift from consuming diets with minimal processed food to consuming highly processed diets (14, 17). Exploring the relationship between type II diabetes and later-life cognitive health in India and China will provide insight into diabetes-related dementia risk in populations that are at their intermediate stages of epidemiologic and nutrition transitions.

In order to assess the relationship between diabetes and cognitive function cross-nationally, cognitive assessments need to be valid across diverse contexts. This includes varying educational, social, cultural and political environments (18). The Harmonized Cognitive Assessment Protocol (HCAP) is a neuropsychological test battery that provides comparable measures across countries through the US Health and Retirement Study (HRS) and its international partner studies (19). Both India and China have ongoing longitudinal aging studies that have administered HCAP to a subset of participants (20, 21). Therefore, India and China have comparable measures that allow for cross-national evaluations to better understand the association between diabetes and cognitive function.

Previous research has investigated the relationship between diabetes and cognitive health in India using data from a partner study, the Longitudinal Aging Study in India – Diagnostic Assessment of Dementia (LASI-DAD) (22). The researchers found that associations were different from findings in high-income countries, where rural respondents with diabetes had greater cognitive scores than respondents without diabetes and urban respondents had similar scores between those with diabetes compared to those without diabetes (22). We seek to build on these findings by comparing the association in India with that observed in China. To achieve this aim, we capitalize on the use of harmonized cognitive assessments, thus allowing for direct comparisons between two countries (18). Based on previous research (22) and differences seen in health patterns between high-income countries and LMICs, we hypothesized that presence of type II diabetes is associated with higher average cognitive scores, rather than lower average cognitive scores typically seen in high-income countries.

## 2 Methods

### 2.1 Study design and sample

This cross-sectional study used harmonized data from two HCAP international partner studies: the Longitudinal Aging Study in India (LASI) (23) and the China Health and Retirement Longitudinal Study (CHARLS) (21, 24). Both LASI and CHARLS are nationally representative studies that collect demographic, health, economic, and social information from participants 45 years and older (along with their spouses) to provide a comprehensive data source on the aging populations of India and China, respectively (21, 23, 24). LASI sampled households using multistage stratified area probability cluster sampling, while CHARLS applied multistage stratified probability sampling of households to randomly select participants (23, 24). Participants who lived in shared living environments, such as military bases or nursing homes, were excluded from the sampling frame for both studies (23, 24). LASI represents 29 states, 6 union territories and 4 metropolitan cities in India (23), while CHARLS is representative of all county units in China, except those in Tibet (24).

Each study administered the HCAP neuropsychological battery to participants 60 years and older, thus establishing corresponding HCAP sub-studies that can undergo cross-national analyses. Participants were informed and agreed to participate in the neuropsychological battery tests before they were administered (20, 25). The LASI-Diagnostic Assessment of Dementia study (LASI-DAD; 2017 to 2019) features harmonized cognitive data from 18 states in India (*N* = 4, 096) (20). These participants were selected based on a two-staged random stratified sampling method (20). Oversampling of participants who are at high-risk of cognitive impairment was also done to identify those with dementia or mild cognitive impairment; further details are available elsewhere (20). In China, the CHARLS Harmonized Cognitive and Dementia Assessment (CHARLS-HCAP; 2018) provides a cross-section of harmonized cognitive data collected during the fourth wave of the CHARLS study (*N* = 9,755) (24). All consenting participants 60 years and older in CHARLS were administered the HCAP neuropsychological battery (25). We excluded 34 (0.8%) participants from the LASI-DAD sample and 14 (0.1%) participants from the CHARLS-HCAP sample with missing data on either the harmonized cognitive outcome (18), diabetes exposure, or covariates of interest. After these exclusions, 4,062 participants from the LASI-DAD sample and 9,741 participants from the CHARLS-HCAP sample were included. Supplementary Figure 1 provides a CONSORT flow chart, depicting the samples used for the analyses.

LASI and LASI-DAD have been approved by the Indian Council of Medical Research (54/01/Indoforeign/Ger/16-NCD-II), the University of Southern California (UP-15-00684), All India Institute of Medical Sciences, New Delhi (IEC-284/06.05.2016, RP-33/ 2016), as well as collaborating institutions (26). CHARLS and CHARLS-HCAP have been approved by the Institutional Review Board at Peking University (Beijing, China; IRB00001052–11015, IRB00001052-11014) (25). Written consent was obtained to participate in the study (25, 26).

### 2.2 Variables

The outcome of interest was cognitive function, determined by a harmonized factor score for general cognitive function (18, 27). This score summarizes HCAP data that collected information on attention, executive function, memory, orientation and verbal fluency (19). Versions of HCAP have been appropriately translated and modified so that instruments are curated to fit the cultural and educational context of each country (27). Pre-statistical and statistical harmonization of cognitive measures were undertaken, details of which have been published (18, 27, 28). Essentially, HCAP testing items were selected if they were deemed comparable based on the expertise of epidemiologists and cultural neuropsychologists (27). An item banking approach using confirmatory factor analysis models then identified cognitive tests that were common across international studies as well as items that were unique to each study (28). A finalized harmonized factor score was developed and tested to have high-reliability and be a valid measure of cognitive function across multiple countries (18).

The primary exposure of interest was type II diabetes status, which was informed by self-reported diabetes status or by objective blood biomarker (HbA1c) data. Participants self-reported whether a doctor or health care professional had ever told them they had diabetes. The presence of diabetes was defined by indicating “yes” via self-report or by having a HbA1c level ≥ 6.5%; HbA1c status overrode in discordant cases. The biomarker cut-off was based on criteria established by the World Health Organization (29) and American Diabetes Association (30).

Sociodemographic covariates were included in multivariable models. They were selected and analyzed based on previous literature, which conducted a similar investigation in India but did not use harmonized factor scores for general cognition (22). Age was analyzed categorically, starting at age 60, (60–64 years, 65–69 years, 70–74 years, 75–79 years, 80–84 years, and 85 years and older). Sex was ascertained by self-report and classified as male or female. Educational attainment in both studies was scaled to the International Standard Classification of Education (ISCED) 2011 standards and categorized into 3 levels: none or early childhood education, less than lower secondary education and upper secondary education or higher (31). Marital status was classified as never married, separated/divorced, widowed, or married/partnered. Smoking status was determined by self-report and categorized as never, former, or current smoker. Designation for area of residence (urban or rural) for LASI-DAD was determined by the Government of India’s Census requirements of urbanicity (23) and for CHARLS-HCAP, this distinction was identified by government-defined urban districts or rural counties in China (24). Abdominal obesity was determined by waist circumference guidelines for India and China as an interviewer-measured waist circumference > 90 cm for men in both countries and a waist circumference > 80 cm or > 85 cm for women in India or China, respectively (32, 33).

### 2.3 Statistical analysis

Descriptive statistics were calculated overall and stratified by type II diabetes status for each HCAP sub-study. Univariable and multivariable linear regression models, with general cognitive function as the outcome, were estimated in coordinated analyses of the LASI-DAD (India) and CHARLS-HCAP (China) samples. A significance level of 0.05 was applied to the multivariable regression models. To facilitate interpretability of results, harmonized variables were used in each sample. Model 1 was an unadjusted regression of general cognitive score on type II diabetes. Model 2 was adjusted for age, sex, educational attainment, marital status, smoking status, area of residence, and abdominal obesity. Model 3 adjusted for Model 2 variables and the interaction between diabetes status and area of residence. Model 4 adjusts for Model 2 variables and an interaction between diabetes status and education attainment.

An exploratory analysis was also conducted on a subset of participants from each sample. This analysis sought to study differences in the association between type II diabetes and cognitive function according to awareness of diabetes status, among participants with confirmed diabetes (HbA1c ≥6.5%). Through this targeted analysis, the implications of a diabetes diagnosis on cognitive function and health can be further understood. We evaluated the average difference in cognitive performance between diagnosed and undiagnosed diabetes, using the previously described regression models. In this secondary analysis, 641 participants from the LASI-DAD sample and 990 participants from the CHARLS-HCAP sample were included (Supplementary Figure 1).

Stata version 17.0 (StataCorp, College Station, Texas) was used for analyses (34). Models were checked for outlying values and high-leverage values using graphical displays of residuals.

## 3 Results

Characteristics of participants with and without type II diabetes, for both samples, are shown in Table 1. The prevalence of type II diabetes in the LASI-DAD sample (*N* = 4,062) was 21.1% (*n* = 858). In CHARLS-HCAP (*N* = 9,741), the prevalence of type II diabetes was 15.6% (*n* = 1,506). LASI-DAD and CHARLS-HCAP samples mainly consist of participants aged 60 to 69 years (57.8 and 62.8%, respectively), were female (54.1 and 50.8%), had no education or early childhood education (62.6 and 50.3%), and resided in rural areas (62.0 and 60.7%). In India, participants with type II diabetes were more likely to live in urban areas (54.9%) compared to those without diabetes (33.5%). This pattern differed in China, where participants with type II diabetes (52.1%) and without (62.3%) resided in rural areas. In both samples, participants with abdominal obesity were more prevalent among participants with diabetes (79.2 and 79.3%).

**Table 1:** Sample characteristics stratified by type II diabetes status for India (2017–2019) and China (2018) Harmonized Cognitive Assessment Protocol (HCAP) study samples (column percentage).

	LASIDAD (India)			CHARLS-HCAP (China)		
	Total (*N* = 4,062)	Diabetes (*n* = 858)	No diabetes (*n* = 3,204)	Total (*N* = 9,741)	Diabetes (*n* = 1,506)	No diabetes (*n* = 8,235)
Age group, *N* (%)						
60–64	1,126 (27.72)	255 (29.72)	871 (27.18)	3,210 (32.95)	455 (30.21)	2,755 (33.45)
65–69	1,222 (30.08)	266 (31.00)	956 (29.84)	2,910 (29.87)	458 (30.41)	2,452 (29.78)
70–74	757 (18.64)	159 (18.53)	598 (18.66)	1,774 (18.21)	293 (19.46)	1,481 (17.98)
75–79	481 (11.84)	101 (11.77)	380 (11.86)	1,101 (11.30)	171 (11.35)	930 (11.29)
80–84	260 (6.40)	38 (4.43)	222 (6.93)	554 (5.69)	101 (6.71)	453 (5.50)
85+	216 (5.32)	39 (4.55)	177 (5.52)	192 (1.97)	28 (1.86)	164 (1.99)
Sex, *N* (%)						
Female	2,196 (54.06)	466 (54.31)	1,730 (54.00)	4,951 (50.83)	864 (57.37)	4,087 (49.63)
Educational attainment^a^, *N* (%)						
None or early childhood	2,541 (62.56)	413 (48.14)	2,128 (66.42)	4,899 (50.29)	712 (47.28)	4,187 (50.84)
Less than lower secondary	836 (20.58)	210 (24.48)	626 (19.54)	3,914 (40.18)	608 (40.37)	3,306 (40.15)
Upper secondary or higher	685 (16.86)	235 (27.39)	450 (14.04)	928 (9.53)	186 (12.35)	742 (9.01)
Marital status, *N* (%)						
Never married	40 (0.98)	4 (0.47)	36 (1.12)	61 (0.63)	6 (0.40)	55 (0.67)
Separated/divorced	33 (0.81)	5 (0.58)	28 (0.87)	116 (1.19)	19 (1.26)	97 (1.18)
Widowed	1,342 (33.04)	257 (29.95)	1,085 (33.86)	1,821 (18.69)	291 (119.32)	1,530 (18.58)
Married/partnered	2,647 (65.16)	592 (69.00)	2,055 (64.14)	7,743 (79.49)	1,190 (79.02)	6,553 (79.57)
Smoking Status, *N* (%)						
Never smoked	3,174 (78.14)	720 (83.92)	2,454 (76.59)	5,303 (54.44)	898 (59.63)	4,405 (53.49)
Former smoker	268 (6.60)	59 (6.88)	209 (6.52)	1,832 (18.81)	311 (20.65)	1,521 (18.47)
Current smoker	620 (15.26)	79 (9.21)	541 (16.89)	2,606 (26.75)	297 (19.72)	2,309 (28.04)
Area of residence, *N* (%)						
Rural	2,517 (61.96)	387 (45.10)	2,130 (66.48)	5,912 (60.69)	784 (52.06)	5,128 (62.27)
Abdominal obesity^b^, *N* (%)						
Absent	1,711 (42.12)	178 (20.75)	1,533 (47.85)	3,590 (36.85)	312 (20.72)	3,278 (39.81)
Present	2,351 (57.88)	680 (79.25)	1,671 (52.15)	6,151 (63.15)	1,194 (79.28)	4,957 (60.19)

LASI-DAD indicates the Longitudinal Aging Study in India – Diagnostic Assessment of Dementia study and CHARLS-HCAP indicates the China Health and Retirement Longitudinal Study. ^a^Education attainment classifications are scaled to ISCED 2011 standards, such that lower secondary indicates US grades 7–9 and upper secondary indicates US grades 10–12. ^b^Presence of abdominal obesity is determined by waist circumference > 90 cm in men for both HCAP study sample, >80 cm in women for LASI-DAD and > 85 cm in women for CHARLS-HCAP.

**Figure 1:**
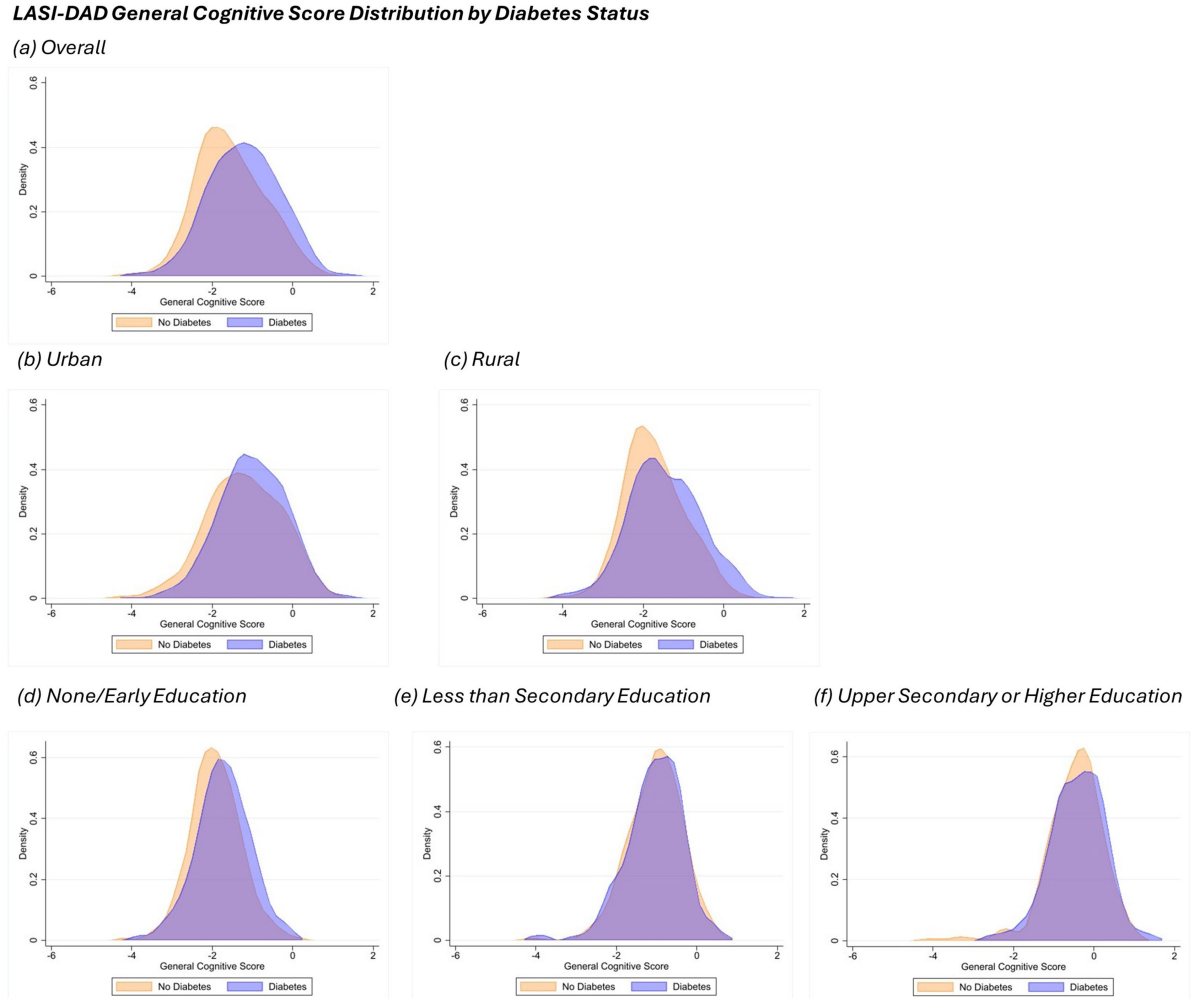
General cognitive score density plot by diabetes status in LASI-DAD. Each panel displays the density distribution of participants in the sample, categorized by diabetes status (orange for No Diabetes and blue for Diabetes). Panel **(a)** indicates the overall sample, panels **(b**,**c)** display plots by area of residence (urban and rural, respectively), while panels **(d**-**f)** indicate density plots by education attainment (none/early, less than lower secondary, and upper secondary or higher). Density plots of general cognitive scores were generated using Epanechnikov kernel density function with bin width of 0.5.

**Figure 2:**
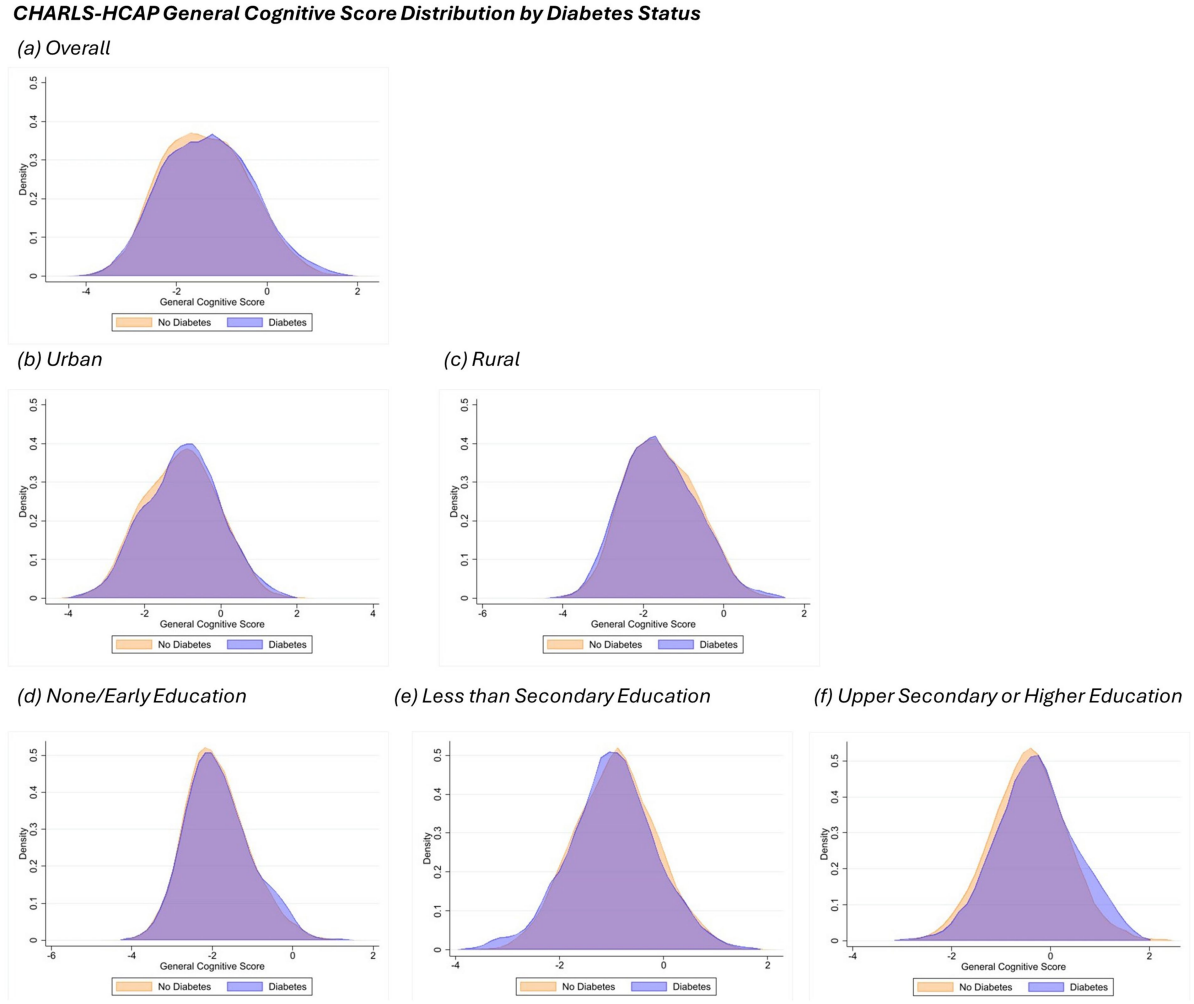
General cognitive score density plot by diabetes status in CHARLS-HCAP. Each panel displays the density distribution of participants in the sample, categorized by diabetes status (orange for No Diabetes and blue for Diabetes). Panel **(a)** indicates the overall sample, panels **(b**,**c)** display plots by area of residence (urban and rural, respectively), while panels **(d**-**f)** indicate density plots by education attainment (none/early, less than lower secondary, and upper secondary or higher). Density plots of general cognitive scores were generated using Epanechnikov kernel density function with bin width of 0.5.

General cognitive score distribution plots by diabetes status, area of residence and educational attainment are shown in Figure 1 for LASI-DAD and Figure 2 for CHARLS-HCAP. For LASI-DAD, the mean general cognitive score among people with diabetes was −1.20 with a standard deviation (SD) of 0.88 and without diabetes was −1.54 (SD: 0.86). For CHARLS, the mean general cognitive score among people with diabetes and without diabetes was −1.30 (SD: 0.98) and −1.36 (SD: 0.96), respectively. For both study samples, participants with diabetes consistently had greater mean general cognitive scores across area of residence groups and education attainment groups compared to participants without diabetes.

### 3.1 LASI-DAD: associations of type II diabetes with cognition

On average, type II diabetes was significantly associated with better general cognitive scores [*β* = 0.340, 95% confidence interval (CI): 0.274 to 0.405] in the unadjusted model (Model 1; Table 2). After adjustment for potential confounders, the association was attenuated to *β* = 0.040, 95% CI: −0.009 to 0.089 (Model 2; Table 2). An interaction term between type II diabetes and area of residence revealed a positive relationship between diabetes and cognitive performance, to those living in rural areas, but not urban areas (Model 3; Table 2). Including an interaction between type II diabetes and educational attainment revealed a positive and statistically significant association between diabetes and general cognition among those with none or early childhood education (*β* = 0.105, 95% CI: 0.038 to 0.171) but not in those with higher education (Model 4; Table 2). The association between diabetes and cognitive scores among participants with less than lower secondary education was significantly weaker than participants with none or early childhood education, although the marginal association in that group was not statistically different from no association.

**Table 2:** Multivariable linear regressions analysis of general cognitive score on type II diabetes by Harmonized Cognitive Assessment Protocol (HCAP) sample for India and China.

LASI-DAD (*N* = 4,062)				
	Model 1	Model 2	Model 3	Model 4
Type II diabetes status				
Diabetes	0.340 [0.274 to 0.405]	0.040 [−0.009 to 0.089]	−0.006 [−0.075 to 0.062]	0.105 [0.038 to 0.171]
Interaction with area of residence				
Diabetes x Rural			0.094 [0.004 to 0.190]	/
Interaction with education attainment				
Diabetes x None or Early Childhood			/	[REF]
Diabetes x Less than Lower Secondary				−0.192 [−0.310 to −0.075]
Diabetes x Upper Secondary or higher				−0.080 [−0.200 to 0.038]
CHARLS-HCAP (*N* = 9,741)				
	Model 1	Model 2	Model 3	Model 4
Type II diabetes status				
Diabetes	0.062 [0.010 to 0.115]	−0.010 [−0.052 to 0.032]	0.012 [−0.050 to 0.074]	0.025 [−0.035 to 0.086]
Interaction with area of residence				
Diabetes x Rural			−0.041 [−0.125 to 0.042]	/
Interaction with education attainment				
Diabetes x None or Early Childhood			/	[REF]
Diabetes x Less than Lower Secondary				−0.120 [−0.208 to −0.030]
Diabetes x Upper Secondary or higher				0.109 [−0.026 to 0.246]

LASI-DAD indicates the Longitudinal Aging Study in India – Diagnostic Assessment of Dementia study and CHARLS-HCAP indicates the China Health and Retirement Longitudinal Study. Estimates reported are regression coefficients for general cognitive score [95% confidence interval]. Model 1 is the unadjusted model. Model 2 is adjusted for age, sex, education attainment, marital status, smoking status, area of residence and abdominal obesity. Model 3 is adjusted for model 2 and interaction between diabetes status and area of residence. Model 4 is adjusted for model 2 and an interaction between diabetes status and education attainment.

Among LASI-DAD participants with HbA1c data (*N* = 2,815) and whose HbA1c level ≥ 6.5% (*N* = 641), 284 (44%) were deemed undiagnosed (they did not self-report they had diabetes) and 357 (56%) self-reported that they had diabetes (Supplementary Table 1). In this subsample, self-report of diabetes was associated with better cognitive performance in the unadjusted model (Model 1; Table 3), but there was no difference after adjustment for confounders (Model 2; Table 3) and no effect modification by area of residence (Model 3; Table 3) or education (Model 4; Table 3).

**Table 3:** Multivariable linear regressions analysis of general cognitive score on type II diabetes diagnosis among participants with HbA1c ≥ 6.5% by Harmonized Cognitive Assessment Protocol (HCAP) sample for India and China.

LASI-DAD (*N* = 641)				
	Model 1	Model 2	Model 3	Model 4
Type II diabetes status				
Self-reported diagnosis^a^	[REF]	[REF]	[REF]	[REF]
Undiagnosed^b^	−0.273 [−0.407 to −0.139]	−0.022 [−0.120 to 0.074]	−0.024 [−0.161 to 0.112]	−0.019 [−0.157 to 0.121]
Interaction with area of residence				
Diabetes x Rural			0.002 [−0.191 to 0.196]	/
Interaction with education attainment				
Diabetes x None or Early Childhood			/	[REF]
Diabetes x Less than Lower Secondary				0.058 [−0.182 to 0.298]
Diabetes x Upper Secondary or higher				−0.071 [−0.306 to 0.164]
CHARLS-HCAP (*N* = 990)				
	Model 1	Model 2	Model 3	Model 4
Type II diabetes status				
Self-reported diagnosis^a^	[REF]	[REF]	[REF]	[REF]
Undiagnosed^b^	0.052 [−0.209 to 0.312]	0.017 [−0.191 to 0.225]	0.214 [−0.085 to 0.515]	−0.124 [−0.435 to 0.186]
Interaction with area of residence				
Diabetes x Rural			−0.378 [−0.793 to 0.037]	/
Interaction with education attainment				
Diabetes x None or Early Childhood			/	[REF]
Diabetes x Less than Lower Secondary				0.302 [−0.142 to 0.747]
Diabetes x Upper Secondary or higher				0.103 [−0.555 to 0.762]

LASI-DAD indicates the Longitudinal Aging Study in India – Diagnostic Assessment of Dementia study and CHARLS-HCAP indicates the China Health and Retirement Longitudinal Study. Estimates reported are regression coefficients for general cognitive score [95% confidence interval]. Model 1 is the unadjusted model. Model 2 is adjusted for age, sex, education attainment, marital status, smoking status, area of residence and abdominal obesity. Model 3 is adjusted for model 2 and interaction between diabetes status and area of residence. Model 4 is adjusted for model 2 and an interaction between diabetes status and education attainment. ^a^Self-reported diagnosis refers to participants who self-reported “yes” to type II diabetes diagnosis and have Hba1c ≥ 6.5%. ^b^Undiagnosed individuals refer to participants who self-reported “no” to type II diabetes diagnosis but have Hba1c ≥ 6.5%.

### 3.2 CHARLS-HCAP: associations of type II diabetes with cognition

Like the LASI-DAD sample, in CHARLS-HCAP a positive significant association between presence of type II diabetes and better cognitive scores was evident in the unadjusted model (Model 1; Table 2). Adjustment for confounders presented a non-significant association in Model 2 (*β* = −0.010, 95% CI: −0.052 to 0.032). There was no evidence of an interaction with area of residence in CHARLS-HCAP (Model 3; Table 2). When we included an interaction term between type II diabetes and educational attainment (Model 4), there was a significant interaction such that diabetes was negatively associated with cognitive score among people with less than lower secondary education only.

Among the 6,717 participants with HbA1c data, the CHARLS-HCAP subsample included 990 participants whose HbA1c level ≥ 6.5%, of whom 54 (5%) were undiagnosed and 936 (95%) self-reported a diagnosis. Within this subsample, there was no association between self-reported diabetes and cognition in either unadjusted or adjusted models (Table 3).

## 4 Discussion

The current study aimed to evaluate the association between type II diabetes and general cognition in India and China, the two largest LMICs. No association was found between type II diabetes and cognition in fully adjusted models for both studies. However, there was a positive association between diabetes and cognition among older adults with none or early childhood education in India. Further investigation determined that area of residence and educational attainment modified the association. In India, participants living in rural areas with diabetes had better cognitive scores compared to those without diabetes. Alternatively, there was no significant difference seen among urban residents. In both India and China, diabetes was associated with worse cognitive scores among participants who have less than lower secondary education compared to those with none/early childhood education. These relationships differ from high income countries. Although further investigation through longitudinal studies is needed, these findings reveal the possible impact of the epidemiologic and nutrition transition in LMICs. Therefore, this study highlights important implications of type II diabetes as a potentially preventable risk factor for late-life cognitive decline in two of the largest LMICs.

Studies on the relationship between type II diabetes and late-life cognition have produced various findings. One study incorporated three cross-sectional surveys, including 10/66 Dementia Research Group survey, Study of global ageing [SAGE], and LASI pilot survey, focused on modifiable risk factors for cognitive function and dementia (35). Similar to our study’s findings, diabetes was associated with better cognitive performance in LASI and SAGE but not in 10/66 data (35). Previous research using CHARLS also ultimately determined no association seen between diabetes and cognition (specifically episodic memory) among participants 60 years and older, after adjusting for cardiovascular factors (4). However, research conducted in Brazil (36) and Mexico (37) have similar associations between type II diabetes and cognition as the relationships seen in high-income countries. Therefore, as research in this area produces differing findings, it is all the more reason to continue investigating this relationship in LMICs.

The differences seen between urban versus rural residents and with varying levels of education in India and China may indicate changes in health patterns that reflect differing stages of the epidemiologic transition and nutrition transition. According to Mattei et al. (14), India and China are both considered countries that are in the ‘ongoing transition’ phase of the epidemiologic transition. This means that like countries in the ‘early transition’ phase, India and China have steep declines in age-adjusted mortality, however they also have higher lifer expectancy and higher median age since these countries possess more modern health care and stronger economic growth (14). During the ‘ongoing transition’ phase, the burden of non-communicable diseases can be predisposed by malnutrition or poor maternal conditions during fetal and early childhood development and then exacerbated through unhealthy lifestyles encouraged by factors, such as urbanization and poor diet, in adulthood (14, 38, 39). Limited health care access and resources further strengthens the burden of non-communicable diseases seen in ‘ongoing transition’ phase countries (14, 40).

In India, area of residence modified the relationship between diabetes and cognitive function, such that the difference in cognitive score between diabetes status is greater for those who live in rural areas compared to those in urban areas. This difference likely reflects the accumulated effect of factors involved in the epidemiologic and nutrition transition effect, as described earlier. Urban residents live in environments that feature economic growth, increased access to highly processed foods, and reduced physical activity, thus encouraging high levels of obesity (14, 41). In addition to a genetic predisposition for type II diabetes in the Indian population (42), living in urban areas may intensify the severity of type II diabetes and related complications, including dementia, thus resulting in worse cognitive outcomes in older adulthood. Additionally, we must consider the influence of access to health care facilities and selection effects. Health care facilities for general health as well as reliable screening and treatment resources for type II diabetes are often unavailable in rural areas (42). Thus, predominant causes of morbidity and mortality in rural areas may reflect what is typically seen in ‘early transition’ countries, where poor sanitation, food insecurity, and communicable diseases are predominant drivers of health (14). This health trend is likely represented in the study sample, as older adult participants with diabetes, especially those who live in rural areas, may be biologically or socioeconomically advantageous, thus having better cognitive outcomes.

An interesting interaction effect between education attainment and diabetes was found in India and China. In both studies, diabetes was associated with better cognitive scores in the none/early childhood education group (significantly so in India) but statistically significantly worse cognitive scores in the group with less than lower secondary education attainment. Based on pattern shifts that are characteristic of the ongoing transitional phase in epidemiological and nutritional transitions (14, 16), participants who have completed up to lower secondary education attainment may be a demographic group who are most vulnerable by the ‘ongoing transition’ phase. These individuals may experience malnutrition or poor health care conditions during early childhood and adolescent years. However, during adulthood they may seek areas with increased employment opportunities, thus encouraging migration to regions with rapid urbanization and engaging in overconsumption of highly refined foods as well as a sedentary lifestyle (43–45). In contrast, older adults who had none or early childhood education likely reside in areas where lifestyles and employment are labor intensive, albeit they likely had less access to health care. While older adults who obtained upper secondary or higher education are indicative of those with higher socioeconomic status, had more access to health care and are associated to have higher late-life cognitive scores (46). Although, these individuals likely sought employment opportunities in urbanized areas that resulted in sedentary behavior and poor diet. Considering the collective effect of risk factors that encourage poor type II diabetes control as well as reduced protective effects of education attainment, general cognition of older adults with less than lower secondary education and diabetes may have poorer outcomes in contrast to those with none or early childhood education attainment. Future directions in research should further investigate these possibilities in India and China.

While associations of type II diabetes with general cognitive performance among participants with confirmed diabetes (HbA1c ≥ 6.5%) were null (Table 3), it does suggest participants who do not self-report a diagnosis have worse general cognitive outcomes compared to those with a diagnosis in India. This may be attributable to limited access to health care and could be manifested by lack of diabetes education and awareness, inaccessible treatments, maintenance of poor lifestyle behaviors, and reduced monitoring of overall health (47). Together these factors could raise the risk of type II diabetes complications, including long-term effects on cognitive impairment and dementia. Furthermore, given that only 5% of the CHARLS-HCAP sample were undiagnosed with type II diabetes and no association was found between self-reported type II diabetes and cognition, this may indicate that the lack of access to health care is less problematic in China compared to India. In comparison to China, the current associations we see in India may be more likely due to disparities in health care access. Whereas the association between type II diabetes and late-life cognitive health may be more attributable to the epidemiologic and nutrition transition in China compared to India. However, we strongly recommend further investigation to confirm these differences, which could inform health care policy and support interventions that address inaccessible health care or improve urban lifestyles within India and China.

There are several strengths to this study. First, we utilized harmonized variables to facilitate more direct comparisons of associations between countries. We also integrated self-report and blood-based biomarker (HbA1c) data to determine presence of type II diabetes, mitigating potential misclassification bias due to undiagnosed individuals. Furthermore, LASI-DAD (48) and CHARLS-HCAP (49) used venous HbA1c samples. In comparison to dried blood spot assays, using venous blood samples allows for more direct result interpretation whereas blood spot samples would require further adjustments for interpretation and various parameters can influence the accuracy of dried blood spot analysis (49, 50). This study was able to deduce associations using nationally representative samples. Both LASI-DAD and CHARLS-HCAP sampled from almost all states/regions in India and China, respectively. Another strength is that LASI-DAD and CHARLS-HCAP studies collected data recently, thus the current study provides updated analyses on type II diabetes status and general cognition.

Despite these strengths, this study also has its limitations. While incorporating blood biomarker data (HbA1c levels) is a strength, a limitation of HbA1c levels is that it can be affected by conditions such as iron deficiency anemia and may skew findings (51). Since cross-sectional data was used, these analyses were unable to capture changes in cognitive function over time, and temporality between the exposure and outcome could not be considered. Furthermore, given that participants had to survive until 60 years-old to participate in the study, this study was unable to establish and compare how the type II diabetes and cognitive score association may differ in younger age groups. In addition, data on diabetes treatments were unavailable or incomplete for many participants.

To build upon our findings, future research is needed to further understand the association between diabetes and cognition using longitudinal cognitive data. Longitudinal studies can incorporate sub-classes of diabetes status (i.e., no diabetes, untreated diabetes, controlled diabetes and treated but uncontrolled diabetes) to assess differences in treatment effects as well as differences in diabetes duration. Given the large number of diverse communities and sub-populations present in these LMICs, future research should also further examine social and health care differences that influence diabetic outcomes and consequently its association with late-life cognitive health. This might include exploring differences between specific geographic areas (ex. hukou systems in China) or by indicators of SES, including caste and household income in addition to education attainment. This will provide additional insight of how the epidemiologic transition and inequalities in health care access, or an education may influence the association between type II diabetes and cognition. Cross-national comparisons can then be determined between socioeconomically similar regions across India and China.

In conclusion, the present study identified overall positive associations between type II diabetes and general cognitive scores in India and China, which deviates from that observed in high-income countries. This difference may indicate ongoing effects of epidemiologic and nutrition transitions seen in both India and China. Area of residence and education attainment modified the relationship between type II diabetes and cognitive function. Urban and rural differences that contribute to the epidemiologic and nutrition transitions, such as urbanization, lifestyle, and possible health care access, may be underlying factors that explain these relationships in India. In both countries, diabetes may have an adverse effect on late-life cognition for socially and financially vulnerable populations who have less than lower secondary education attainment. Future longitudinal research is recommended to determine the underlying factors of these relationships in order to inform policy makers, public health interventions and educators on mitigating the risk of cognitive decline in older adults with diabetes.

## Data Availability

Publicly available datasets were analyzed in this study. This data can be found at: Gateway of Global Aging Data platform: https://g2aging.org/hrd/overview.

## References

[1] 2022 Alzheimer’s disease facts and figures. Alzheimers Dement. (2022) 18:700–89. doi: 10.1002/alz.12638 35289055

[2] WHO. Global status report on the public health response to dementia. (2021). Available at: https://www.who.int/publications/i/item/9789240033245. Accessed July 25, 2024

[3] Norton S Matthews FE Barnes DE Yaffe K Brayne C. Potential for primary prevention of Alzheimer’s disease: an analysis of population-based data. Lancet Neurol. (2014) 13:788–94. doi: 10.1016/S1474-4422(14)70136-X 25030513

[4] Zhang L Yang J Liao Z Zhao X Hu X Zhu W. Association between diabetes and cognitive function among people over 45 years old in China: a cross-sectional study. Int J Environ Res Public Health. (2019) 16:1294. doi: 10.3390/ijerph16071294, PMID: 30978913 PMC6479487

[5] Li X Song D Leng SX. Link between type 2 diabetes and Alzheimer’s disease: from epidemiology to mechanism and treatment. Clin Interv Aging. (2015) 10:549–60. doi: 10.2147/CIA.S74042, PMID: 25792818 PMC4360697

[6] Ryan JP Fine DF Rosano C. Type 2 diabetes and cognitive impairment: contributions from neuroimaging: contributions from neuroimaging. J Geriatr Psychiatry Neurol. (2014) 27:47–55. doi: 10.1177/0891988713516543, PMID: 24394151 PMC4049175

[7] Gudala K Bansal D Schifano F Bhansali A. Diabetes mellitus and risk of dementia: a meta-analysis of prospective observational studies. J Diabetes Investig. (2013) 4:640–50. doi: 10.1111/jdi.12087 PMC4020261 24843720

[8] Takeda S Sato N Rakugi H Morishita R. Molecular mechanisms linking diabetes mellitus and Alzheimer disease: beta-amyloid peptide, insulin signaling, and neuronal function. Mol BioSyst. (2011) 7:1822–7. doi: 10.1039/c0mb00302f, PMID: 21431241

[9] Liu J Wang L-N Tan J-P. Dementia in China: current status. Neurology. (2013) 81:1077–8. doi: 10.1212/WNL.0b013e3182a4a3cb, PMID: 24042573

[10] Ravindranath V Sundarakumar JS. Changing demography and the challenge of dementia in India. Nat Rev Neurol. (2021) 17:747–58. doi: 10.1038/s41582-021-00565-x, PMID: 34663985 PMC8522537

[11] Magliano D Boyko EJ. IDF Diabetes Atlas. Brussels, Belgium: International Diabetes Federation (2021). 35914061

[12] Liu X Zhang L Chen W. Trends in economic burden of type 2 diabetes in China: based on longitudinal claim data. Front Public Health. (2023) 11:1062903. doi: 10.3389/fpubh.2023.1062903, PMID: 37143967 PMC10151735

[13] Popkin BM. Nutrition transition and the global diabetes epidemic. Curr Diab Rep. (2015) 15:64. doi: 10.1007/s11892-015-0631-4, PMID: 26209940 PMC4942180

[14] Mattei J Malik V Wedick NM Hu FB Spiegelman D Willett WC. Global nutrition epidemiologic transition initiative. Reducing the global burden of type 2 diabetes by improving the quality of staple foods: the global nutrition and epidemiologic transition initiative. Glob Health. (2015) 11:23. doi: 10.1186/s12992-015-0109-9, PMID: 26040275 PMC4489001

[15] Omran AR. The epidemiologic transition: a theory of the epidemiology of population change. Milbank Mem Fund Q. (1971) 49:509. doi: 10.2307/3349375 5155251

[16] Mattei J Malik V Wedick NM Campos H Spiegelman D Willett W. A symposium and workshop report from the global nutrition and epidemiologic transition initiative: nutrition transition and the global burden of type 2 diabetes. Br J Nutr. (2012) 108:1325–35. doi: 10.1017/S0007114512003200, PMID: 22863082

[17] Popkin BM. Global nutrition dynamics: the world is shifting rapidly toward a diet linked with noncommunicable diseases 1–3. Am J Clin Nutr. (2006) 84:289–98. doi: 10.1093/ajcn/84.1.289, PMID: 16895874

[18] Gross AL Li C Briceño EM Arce Rentería M Jones RN Langa KM. Harmonisation of later-life cognitive function across national contexts: results from the harmonized cognitive assessment protocols. Lancet Healthy Longev. (2023) 4:e573–83. doi: 10.1016/s2666-7568(23)00170-8, PMID: 37804847 PMC10637129

[19] Langa KM Ryan LH McCammon RJ Jones RN Manly JJ Levine DA. The health and retirement study harmonized cognitive assessment protocol project: study design and methods. Neuroepidemiology. (2020) 54:64–74. doi: 10.1159/000503004, PMID: 31563909 PMC6949364

[20] Banerjee J Jain U Khobragade P Weerman B Hu P Chien S. Methodological considerations in designing and implementing the harmonized diagnostic assessment of dementia for longitudinal aging study in India (LASI-DAD). Biodemography Soc Biol. (2020) 65:189–213. doi: 10.1080/19485565.2020.1730156, PMID: 32727279 PMC7398273

[21] Chen X Wang Y Strauss J Zhao Y. China health and retirement longitudinal study (CHARLS) In: Gu D Dupre ME, editors. Encyclopedia of gerontology and population aging. Cham: Springer International Publishing (2019). 1–9.

[22] Flood D Green H Gross AL Kobayashi LC Levine DA Lee J. iabetes and cognitive health in India: A nationally representative survey of adults aged 45 years and older. medRxiv. (2022) doi: 10.1101/2022.10.14.22281097 PMC12278164 40685236

[23] Perianayagam A Bloom D Lee J Parasuraman S Sekher TV Mohanty SK. Cohort profile: the longitudinal ageing study in India (LASI). Int J Epidemiol. (2022) 51:e167–76. doi: 10.1093/ije/dyab266, PMID: 35021187 PMC9365624

[24] Zhao Y Hu Y Smith JP Strauss J Yang G. Cohort profile: the China health and retirement longitudinal study (CHARLS). Int J Epidemiol. (2014) 43:61–8. doi: 10.1093/ije/dys203, PMID: 23243115 PMC3937970

[25] Zhao Y Strauss J Chen X Wang Y Gong J Meng Q. China health and retirement longitudinal study wave 4 User’s guide. (2020).

[26] LASI-DAD Study Collaborators. Harmonized diagnostic assessment of dementia for the longitudinal aging study in India (LASI-DAD) wave 1 report University of Southern California, USA: Program on Global Aging, Health, and Policy, Center for Economic and Social Research (2002).

[27] Briceño EM Arce Rentería M Gross AL Jones RN Gonzalez C Wong R. A cultural neuropsychological approach to harmonization of cognitive data across culturally and linguistically diverse older adult populations. Neuropsychology. (2023) 37:247–57. doi: 10.1037/neu0000816, PMID: 35482625 PMC9639608

[28] Vonk JMJ Gross AL Zammit AR Bertola L Avila JF Jutten RJ. Cross-national harmonization of cognitive measures across HRS HCAP (USA) and LASI-DAD (India). PLoS One. (2022) 17:e0264166. doi: 10.1371/journal.pone.0264166, PMID: 35213581 PMC8880818

[29] World Health Organisation Consultation. Use of glycated haemoglobin (HbA1c) in the diagnosis of diabetes mellitus. Diabetes Res Clin Pract. (2011) 93:299–309. doi: 10.1016/j.diabres.2011.03.012 21820751

[30] American Diabetes Association. 2. Classification and diagnosis of diabetes: standards of medical care in diabetes-2021. Diabetes Care. (2021) 44:S15–33. doi: 10.2337/dc21-S002, PMID: 33298413

[31] Goldstone L. An international standard classification of education (ISCED). Prospects. (1973) 3:390–7. doi: 10.1007/bf02198536

[32] Misra A Chowbey P Makkar BM Vikram NK Wasir JS Chadha D. Consensus statement for diagnosis of obesity, abdominal obesity and the metabolic syndrome for Asian Indians and recommendations for physical activity, medical and surgical management. J Assoc Physicians India. (2009) 57:163–70. PMID: 19582986

[33] Bao Y Lu J Wang C Yang M Li H Zhang X. Optimal waist circumference cutoffs for abdominal obesity in Chinese. Atherosclerosis. (2008) 201:378–84. doi: 10.1016/j.atherosclerosis.2008.03.001 18417137

[34] StataCorp. Stata statistical software: Release 17. College Station, TX: StataCorp LLC (2021).

[35] Belessiotis-Richards C Livingston G Marston L Mukadam N. A cross-sectional study of potentially modifiable risk factors for dementia and cognitive function in India: a secondary analysis of 10/66, LASI, and SAGE data. Int J Geriatr Psychiatry. (2021) 37:1–13. doi: 10.1002/gps.5661, PMID: 34808698

[36] Teixeira MM Passos VMA Barreto SM Schmidt MI Duncan BB Beleigoli AMR. Association between diabetes and cognitive function at baseline in the Brazilian longitudinal study of adult health (ELSA- Brasil). Sci Rep. (2020) 10:1596. doi: 10.1038/s41598-020-58332-9, PMID: 32005901 PMC6994611

[37] Avila JC Mejia-Arangom S Jupiter D Downer B Wong R. The effect of diabetes on the cognitive trajectory of older adults in Mexico and the United States. J Gerontol B Psychol Sci Soc Sci. (2021) 76:e153–64. doi: 10.1093/geronb/gbaa094, PMID: 32678911 PMC7955990

[38] Amuna P Zotor FB. Epidemiological and nutrition transition in developing countries: impact on human health and development: the epidemiological and nutrition transition in developing countries: evolving trends and their impact in public health and human development. Proc Nutr Soc. (2008) 67:82–90. doi: 10.1017/s0029665108006058, PMID: 18234135

[39] Dandona L Dandona R Kumar GA Shukla DK Paul VK Balakrishnan K. Nations within a nation: variations in epidemiological transition across the states of India, 1990–2016 in the global burden of disease study. Lancet. (2017) 390:2437–60. doi: 10.1016/s0140-6736(17)32804-0, PMID: 29150201 PMC5720596

[40] Schmidhuber J Shetty P. The nutrition transition to 2030: why developing countries are likely to bear the burden. (2005), 2, 150–166, Taylor & Francis: Basingstoke

[41] Paul S Paul S. Transition in dietary quality: evidence from India. Br J Nutr. (2022) 129:2054–66. doi: 10.1017/S0007114522002847, PMID: 36068713

[42] Kaveeshwar SA Cornwall J. The current state of diabetes mellitus in India. Australas Med J. (2014) 7:45–8. doi: 10.4066/AMJ.2014.1979 24567766 PMC3920109

[43] Davies AA Borland RM Blake C West HE. The dynamics of health and return migration. PLoS Med. (2011) 8:e1001046. doi: 10.1371/journal.pmed.1001046, PMID: 21738448 PMC3124523

[44] Shahul Hameed S Kutty VR Vijayakumar K Kamalasanan A. Migration status and prevalence of chronic diseases in Kerala state, India. Int J Chronic Dis. (2013) 2013:1–6. doi: 10.1155/2013/431818, PMID: 26464844 PMC4590921

[45] He H Zhang J Xiu D. China’s migrant population and health. China Popul Dev Stud. (2019) 3:53–66. doi: 10.1007/s42379-019-00032-7

[46] Zhang YS O’Shea B Yu X Cho T-C Zhang KP Kler J. Educational attainment and later-life cognitive function in high- and middle-income countries: evidence from the harmonized cognitive assessment protocol. J Gerontol B Psychol Sci Soc Sci. (2024) 79. doi: 10.1093/geronb/gbae005, PMID: 38284333 PMC10997278

[47] Garg R. Diabetes education & prevention. Indian J Med Res. (2013) 138:820–3. PMID: 24521619 PMC3978965

[48] Lee J Banerjee J Khobragade PY Angrisani M Dey AB. LASI-DAD study: a protocol for a prospective cohort study of late-life cognition and dementia in India. BMJ Open. (2019) 9:e030300. doi: 10.1136/bmjopen-2019-030300, PMID: 31371300 PMC6677961

[49] Chen X Crimmins E Hu PP Kim JK Meng Q Strauss J. Venous blood-based biomarkers in the China health and retirement longitudinal study: rationale, design, and results from the 2015 wave. Am J Epidemiol. (2019) 188:1871–7. doi: 10.1093/aje/kwz170, PMID: 31364691 PMC6825825

[50] Mastronardi CA Whittle B Tunningley R Neeman T Paz-Filho G. The use of dried blood spot sampling for the measurement of HbA1c: a cross-sectional study. BMC Clin Pathol. (2015) 15:13. doi: 10.1186/s12907-015-0013-5, PMID: 26157353 PMC4495815

[51] Lim W-Y Ma S Heng D Tai ES Khoo CM Loh TP. Screening for diabetes with HbA1c: test performance of HbA1c compared to fasting plasma glucose among Chinese, Malay and Indian community residents in Singapore. Sci Rep. (2018) 8:12419. doi: 10.1038/s41598-018-29998-z, PMID: 30127499 PMC6102285

