# Supplementary material for: Type II diabetes and cognitive function among older adults in India and China—results from Harmonized Cognitive Assessment Protocol studies
Source: Front Public Health. 2024 Nov 6;12:1474593. doi: 10.3389/fpubh.2024.1474593 (PMC11576311; doi:10.3389/fpubh.2024.1474593)
Supplement: Supplementary file 1 [file Table_1.docx]

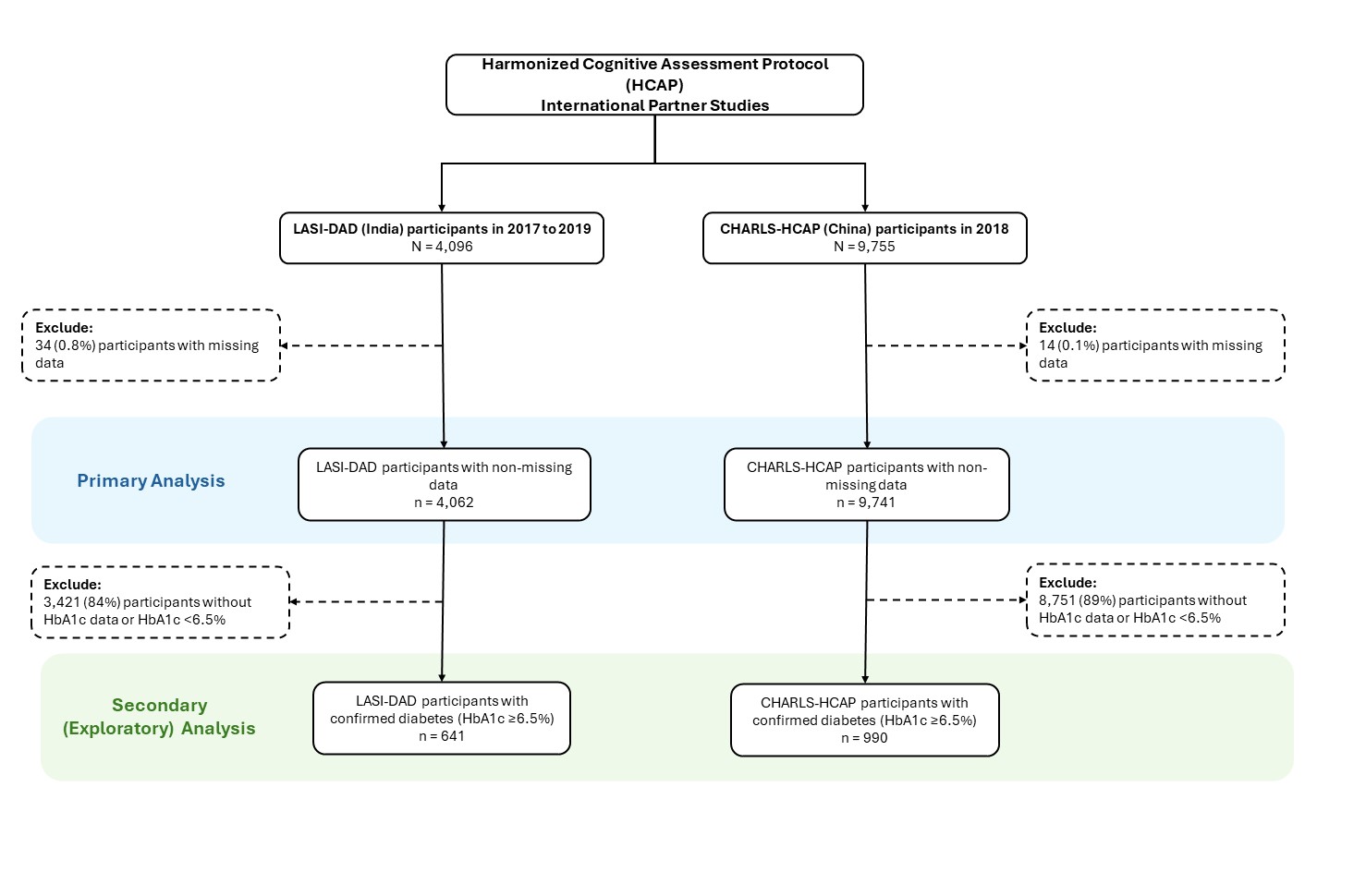


**Supplementary Figure 1.** The CONSORT flow diagram provides an overview of both LASI-DAD (India) and CHARLS-HCAP (China) study sample inclusion criteria for primary and secondary (exploratory) analyses. LASI-DAD indicates the Longitudinal Aging Study in India – Diagnostic Assessment of Dementia study and CHARLS-HCAP indicates the China Health and Retirement Longitudinal Study.

| **Supplementary Table 1 – Sample characteristics stratified by type II diabetes diagnosis status among participants with** **HbA1c ≥ 6.5% for LASI-DAD (2017-2019) and CHARLS-HCAP (2018) (column percentage)** | | | | | | | |
| --- | --- | --- | --- | --- | --- | --- | --- |
|  | LASIDAD  (India) | | |  | CHARLS-HCAP  (China) | | |
|  | Total  (N = 641) | Undiagnosed ^c^  (n = 284) | Self-Reported ^d^  (n = 357) |  | Total  (N = 990) | Undiagnosed ^c^  (n = 54) | Self-Reported ^d^  (n = 936) |
| **Age group, N (%)** | | | | | | | |
| 60-64 | 197 (30.73) | 90 (31.69) | 107 (29.97) |  | 287 (28.99) | 19 (35.19) | 268 (28.63) |
| 65-69 | 200 (31.20) | 83 (29.23) | 117 (32.77) |  | 305 (30.81) | 17 (31.48) | 288 (30.77) |
| 70-74 | 119 (18.56) | 49 (17.25) | 70 (19.61) |  | 202 (20.40) | 7 (12.96) | 195 (20.83) |
| 75-79 | 73 (11.39) | 32 (11.27) | 41 (11.48) |  | 113 (11.41) | 6 (11.11) | 107 (11.43) |
| 80-84 | 27 (4.21) | 13 (4.58) | 14 (3.92) |  | 64 (6.46) | 4 (7.41) | 60 (6.41) |
| 85+ | 25 (3.90) | 17 (5.99) | 8 (2.24) |  | 19 (1.92) | 1 (1.85) | 18 (1.92) |
| **Sex, N (%)** | | | | | | | |
| Female | 343 (53.51) | 158 (55.63) | 185 (51.82) |  | 583 (58.89) | 31 (57.41) | 552 (58.97) |
| **Educational Attainment ^a^, N (%)** | | | | | | | |
| None or Early Childhood | 309 (48.21) | 166 (58.45) | 143 (40.06) |  | 490 (49.49) | 24 (44.44) | 466 (49.79) |
| Less than Lower Secondary | 156 (24.34) | 59 (20.77) | 97 (27.17) |  | 400 (40.40) | 23 (42.59) | 377 (40.28) |
| Upper Secondary or higher | 176 (27.46) | 59 (20.77) | 117 (32.77) |  | 100 (10.10) | 7 (12.96) | 93 (9.94) |
| **Marital Status, N (%)** | | | | | | | |
| Never Married | 4 (0.62) | 1 (0.35) | 3 (0.84) |  | 5 (0.51) | 0 (0.00) | 5 (0.53) |
| Separated/Divorced | 5 (0.78) | 2 (0.70) | 3 (0.84) |  | 7 (0.71) | 0 (0.00) | 7 (0.75) |
| Widowed | 181 (28.24) | 94 (33.10) | 87 (24.37) |  | 188 (18.99) | 15 (27.78) | 173 (18.48) |
| Married/Partnered | 451 (70.36) | 187 (65.85) | 264 (73.95) |  | 790 (79.80) | 39 (72.22) | 751 (80.24) |
| **Smoking Status, N (%)** | | | | | | | |
| Never Smoked | 528 (82.37) | 224 (78.87) | 304 (85.15) |  | 590 (59.60) | 36 (66.67) | 554 (59.19) |
| Former Smoker | 47 (7.33) | 26 (9.15) | 21 (5.88) |  | 209 (21.11) | 4 (7.41) | 205 (21.90) |
| Current Smoker | 66 (10.30) | 34 (11.97) | 32 (8.96) |  | 191 (19.29) | 14 (25.93) | 177 (18.91) |
| **Area of Residence, N (%)** | | | | | | | |
| Rural | 311 (48.52) | 165 (58.10) | 146 (40.90) |  | 549 (55.45) | 28 (51.85) | 521 (55.66) |
| **Abdominal Obesity ^b^, N (%)** |  |  |  |  |  |  |  |
| Present | 311 (48.52) | 165 (58.10) | 146 (40.90) |  | 748 (75.56) | 39 (72.22) | 709 (75.75) |
| LASI-DAD indicates the Longitudinal Aging Study in India – Diagnostic Assessment of Dementia study and CHARLS-HCAP indicates the China Health and Retirement Longitudinal Study. ^a^ Education attainment classifications are scaled to ISCED 2011 standards, such that lower secondary indicates US grades 7-9 and upper secondary indicates US grades 10-12. ^b^ Presence of abdominal obesity is determined by waist circumference >90cm in men for both HCAP study sample, >80cm in women for LASI-DAD and >85cm in women for CHARLS-HCAP.^c^ Undiagnosed individuals refer to participants who self-reported “no” to type II diabetes diagnosis but have Hba1c ≥ 6.5%. ^d^ Self-reported diagnosis refers to participants who self-reported “yes” to type II diabetes diagnosis and have Hba1c ≥ 6.5%. Longitudinal Aging Study in India – Diagnostic Assessment of Dementia is represented as LASI-DAD and China Health and Retirement Longitudinal Study Harmonized Cognitive Assessment Protocol is represented as CHARLS-HCAP. | | | | | | | |
